# Silver Nanoparticle-Mediated Cellular Responses in Various Cell Lines: An in Vitro Model

**DOI:** 10.3390/ijms17101603

**Published:** 2016-09-22

**Authors:** Xi-Feng Zhang, Wei Shen, Sangiliyandi Gurunathan

**Affiliations:** 1 College of Biological and Pharmaceutical Engineering, Wuhan Polytechnic University, Wuhan 430023, China; zhangxf9465@163.com; 2 Key Laboratory of Animal Reproduction and Germplasm Enhancement in Universities of Shandong, College of Animal Science and Technology, Qingdao Agricultural University, Qingdao 266109, China; shenwei427@163.com; 3 Department of Stem Cell and Regenerative Biotechnology, Konkuk University, Seoul 143-701, Korea

**Keywords:** silver nanoparticles, cellular effect, epithelial cells, endothelial cells, macrophage, keratinocytes, fibroblasts, neuronal cells, stem cells

## Abstract

Silver nanoparticles (AgNPs) have attracted increased interest and are currently used in various industries including medicine, cosmetics, textiles, electronics, and pharmaceuticals, owing to their unique physical and chemical properties, particularly as antimicrobial and anticancer agents. Recently, several studies have reported both beneficial and toxic effects of AgNPs on various prokaryotic and eukaryotic systems. To develop nanoparticles for mediated therapy, several laboratories have used a variety of cell lines under in vitro conditions to evaluate the properties, mode of action, differential responses, and mechanisms of action of AgNPs. In vitro models are simple, cost-effective, rapid, and can be used to easily assess efficacy and performance. The cytotoxicity, genotoxicity, and biocompatibility of AgNPs depend on many factors such as size, shape, surface charge, surface coating, solubility, concentration, surface functionalization, distribution of particles, mode of entry, mode of action, growth media, exposure time, and cell type. Cellular responses to AgNPs are different in each cell type and depend on the physical and chemical nature of AgNPs. This review evaluates significant contributions to the literature on biological applications of AgNPs. It begins with an introduction to AgNPs, with particular attention to their overall impact on cellular effects. The main objective of this review is to elucidate the reasons for different cell types exhibiting differential responses to nanoparticles even when they possess similar size, shape, and other parameters. Firstly, we discuss the cellular effects of AgNPs on a variety of cell lines; Secondly, we discuss the mechanisms of action of AgNPs in various cellular systems, and try to elucidate how AgNPs interact with different mammalian cell lines and produce significant effects; Finally, we discuss the cellular activation of various signaling molecules in response to AgNPs, and conclude with future perspectives on research into AgNPs.

## 1. Introduction

The nanotechnology industry is growing rapidly and is able to create novel nanoscale products (1–100 nm) with significant and exciting physical and chemical properties [1,2]. Silver nanoparticles (AgNPs) are one of the fastest-growing nanomaterial categories for consumer, industrial, and biomedical applications owing to their unique properties including high electrical and optical conductivity, surface-enhanced Raman scattering, chemical stability, catalytic activity, particularly enhanced surface area ratios, and separation in electronic energy levels [3,4]. Consequently, AgNPs have been used extensively for biomedical applications including antibacterial, antifungal, antiviral, anti-angiogenic, antitumor, biosensors, and bioimaging.

Despite their demonstrated benefits, recent studies have reported on the toxicological effects of AgNPs under certain conditions. In particular, toxic effects in different cell types depend on the interactions and distribution patterns of the nanoparticles [5]. The size of nanoparticles influences the binding and activation of membrane receptors and subsequent protein expression in cancer cells [6]. For example, AshaRani et al. [6] reported that AgNPs are capable of adsorbing cytosolic proteins on their surface, which may influence the function of intracellular factors that regulate genes involved in DNA damage, cell cycle progression, and DNA damage response/repair in cancer cell lines. Cancer commonly develops in the connective tissue, particularly from epithelial cells, accounting for around 80%–90%. Several studies have addressed the effect of AgNPs on various types of cancer cells including those of the breast, lung, and ovary. A549 cells, derived from human lung carcinoma, are widely used to study cytotoxicity, reactive oxygen species (ROS) generation, oxidative stress, and the molecular mechanisms of apoptosis [7,8,9]. Several studies have reported that AgNPs induce genotoxicity and cytotoxicity in both cancer and normal cell lines [10], alter cell morphology [11], reduce cell viability [12], and cause oxidative stress in lung fibroblasts, glioblastoma cells [13], and human breast cancer cells [14]. AgNP-induced cytotoxicity reduces cell viability in various cell lines by causing apoptosis through the mitochondrial pathway [7,10] and ROS generation [15].

The endothelium is an important target for drug and gene therapy [16]. The integrity of the blood–brain barrier is important for cell survival, and alteration of permeability is involved in the pathogenesis of various cardiovascular diseases, inflammation, acute lung injury syndromes, and carcinogenesis [17]. Tight junctions (TJs) are essential for maintaining the proper environment for neuronal function and establishing the barrier between endothelial cells in retinal and brain blood vessels [18]. With respect to the blood–brain barrier (BBB) function and the neurotoxic response of AgNPs, previous studies suggest that AgNPs can easily cross the BBB and damage barrier integrity by altering endothelial cell membrane permeability [19].

Following AgNP entry into the human body, they not only reach and affect organs such as the lung, liver, spleen, and kidney but also target the central nervous system (CNS) [20,21]. Interestingly, Panyala et al. [22] reported that various organs can be freed from AgNPs after a prolonged period; however, these AgNPs exhibit a longer half-life within the brain than in other organs. They can also easily access the brain by traversing the blood–brain barrier [23]. Therefore, there are potential health risks associated with AgNP exposure. Several in vitro studies have addressed these issues. For instance, Haase et al. [24] exposed mixed primary neuronal cell cultures from the mouse frontal cortex with AgNPs and found that they induced an acute intracellular calcium rise followed by a strong oxidative stress response. Recently, Xu et al. [25] reported that AgNPs induced toxicity and neuronal cell death in primary rat cortical cell cultures, via modulation of cytoskeleton components, perturbations of pre- and postsynaptic proteins, and mitochondrial dysfunction. Dayem et al. [26] found that AgNPs could promote neuronal differentiation of SH-SY5Y cells through increased intracellular ROS generation, enhanced activation of kinases such as protein kinase B (PKB), also known as Akt, is a serine/threonine-specific protein kinase and Erk1/2, and modulation of expression levels of dual-specificity phosphatase (DUSP) genes.

The effect of AgNPs on development remains obscure, despite the investigation of toxicity in germ cells, germline stem cells (GSCs), and somatic cyst stem cells (CySCs) Several studies have shown that various mesoporous silica and magnetic nanoparticles (NPs) have no significant effects on the morphology, proliferation, viability, and differentiation capacity of stem cells [27]. However, only a few studies have reported the effect of AgNPs on human mesenchymal and other stem cells.

Nanoparticles translocate into cells through diffusion, transmembrane channels, or adhesive interactions, which is controlled by several factors including the surface charge of the nanoparticles, particle types, and sizes. Size is the most important factor for cell modification [28]. Not surprisingly, the cytotoxicity of nanoparticles is dependent on various factors such as size, surface charge, coating, particle aggregation, and cell type [29]. In vitro studies are convenient for evaluating all these factors. In vitro models are used frequently to evaluate the effect of drugs or toxic agents, including AgNPs, on bacterial or eukaryotic cells and provide data for analytical studies [30]. In vitro models have been used to assess the efficacy of antibacterial, anticancer, antiviral, and anti-inflammatory AgNP properties. There are two basic study types performed using in vitro model systems: descriptive studies that evaluate the effect of a simulated human dose; and analytic studies that investigate the pharmacodynamics of a particular agent [30]. Sometimes in vitro studies are better than in vivo studies in order to assess bioequivalence (BE) of immediate-release (IR) solid oral dosage forms. In addition, in vitro studies are cost-effective, allow direct assessment of the products, and are free from ethical considerations [31]. In vitro models can provide data on the properties of agents such as nanoparticles; however, the results should be viewed within the context of human and animal data. Although in vitro studies are, by definition, artificial, they are basic and important studies for providing valuable data for assessing potential risks to humans [32]. Cell culture models are used to screen toxicity by evaluating the basal and specialized functions of the cell. Nevertheless, the in vivo model is essential for validating the conclusions reached using in vitro models. Therefore, we have chosen to examine the differential cellular effects of AgNPs in in vitro studies using various cell lines. The primary objective of this review is to summarize the effects of AgNPs on various eukaryotic cells, including their morphology and biological functions. The second objective is to examine how AgNPs interact with different mammalian cell lines, and elucidate the mechanisms of action of AgNPs in various cellular systems.

## 2. Cellular Effects of Silver Nanoparticles (AgNPs) on Epithelial Cells

**Figure 1:**
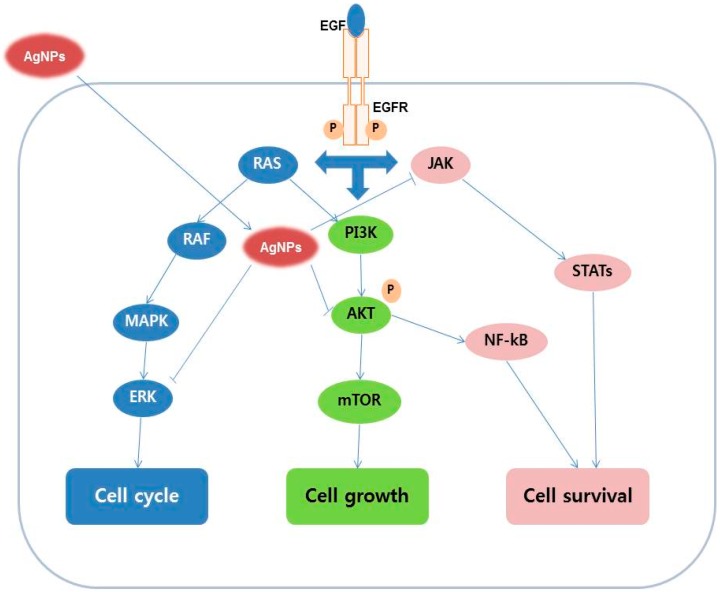
Proposed possible signaling pathways of inhibition effect of silver nanoparticles on EGF-induced cell growth, cell cycle, and cell survival in epithelial cancerous cells. AgNPs, silver nanoparticles; EGFR, epidermal growth factor receptor; RAS, Ras is a membrane-associated guanine nucleotide-binding protein; RAF, RAF kinases are a family of three serine/threonine-specific protein kinases; MAPK, mitogen-activated protein kinases; ERK, extracellular-signal-regulated kinases; PI3K, phosphatidylinositide 3-kinases; AKT, also known as protein kinase B (PKB); mTOR, mechanistic target of rapamycin; JAK, Janus kinase; STATs, signal transducers and activators of transcription; NF-κB, nuclear factor-kappa B.

Treatment of Human Chang liver (HeLa) cells with AgNPs induces cell growth and morphological changes, oxidative cell damage via mitochondria-mediated apoptosis, and modulation of the level of reduced glutathione in the cells [33]. When lung epithelial cells were exposed to AgNPs cell viability was reduced, leakage of lactate dehydrogenase (LDH) was increased, cell cycle distribution was altered, apoptotic gene expression was upregulated, and anti-apoptotic genes were downregulated [34]. When A549 cells were exposed to AgNPs at 10, 50, and 200 μg/mL for 24 h, the cell morphology were significantly altered, which is a characteristic feature of cell death, including cell shrinkage, few cellular extensions, restricted spreading pattern, and increased floating cells [34]. During exposure of a normal human lung bronchial epithelial cell line (BEAS-2B) to AgNPs at different concentrations between 0.01 and 10 μg/mL for 24 h, internalization of AgNPs into the cells as aggregates encased in endocytic vesicles was seen, ultimately causing genotoxic effects with increased ROS generation, formation of micronucleus, and enhanced DNA damage [35]. Subsequently, Comfort et al. [36] elucidated the endocytic mechanism of entry of AgNPs into epithelial cells and the localization of AgNPs in intracellular vacuoles. In addition, AgNPs inhibited epidermal growth factor (EGF) dependent signal transduction through production of high ROS levels, and reduced Akt and ERK signaling. Figure 1 shows a proposed schematic diagram of AgNP-induced apoptosis mechanisms in epithelial cells.

When the cells were exposed to 5 μg/mL AgNPs and 5 and 25 μg/mL gold nanoparticles (AuNPs) and superparamagnetic iron oxide nanoparticles (SPION), regardless of composition, the nanoparticles reduced EGF-dependent phosphorylation levels of Akt (p-Akt) and (p-Erk). However, AuNPs had the most significant impact, with an observed 15% inhibition of p-Akt combined with a 30%–40% reduction of p-Erk. Similarly, AgNPs Inhibited both p-Akt and p-Erk by approximately 20% [36]. A study was performed to determine how the aggregation of silver influences the kinetics of cellular binding and uptake and induction of cytotoxic responses in human alveolar epithelial (A549), hepatic (HepG2), and undifferentiated monocyte (THP-1) cell lines. The aggregation of silver is entirely dependent on size, time point, and cell type. Each cell line exhibited unique behavior. The A549 cells were exposed to 50 μg/mL of Ag particles (20 or 200 nm) for 24 h; 20 nm AgNPs were visible as single nanoparticles and agglomerates distributed mainly in the cytoplasm. A549 cells were the most sensitive to cellular binding and uptake. HepG2 cells showed increased sensitivity in relation to metabolic activation, and THP-1 cells were the most resistant. This indicates that agglomeration, particle size, and cell type are critical factors for cytotoxicity [37]. Differences in cellular binding/uptake and sensitivity was tested using different concentration of Ag particles including 10, 50 and 100 μg/mL for 2, 24, 48, and 72 h. A549 cells were most prone to binding/uptake of the 2 nm AgNPs. Another study from Stoehr and coworkers [38] demonstrated the cytotoxic effect of AgNP shape on A549 cells. Surprisingly, spherical particles had no effect on cell viability, LDH release, cytokine promoter induction, and nuclear factor-kappa B (NF-κB) activation, whereas nanowires had a significant impact on all of these parameters. When the cells were exposed to higher silver wire concentrations (c0/4 and higher, 2.25 × 10^9^–1.35 × 10^10^ Wrs/mL, 9 × 10^15^–1.01 × 10^16^ nm²/mL, 3.68–3.83 mg/mL), enhanced amounts of LDH were released by naïve cells and the effect was most pronounced for the smaller wires. This study proves that toxicity is dependent on particle shape and not the release of silver ions [38].

Surface coating is another important factor for toxicity. To demonstrate the effect of surface coating Suresh et al. [29] investigated the toxicity of AgNPs in epithelial cells. They concluded that toxicity not only depends on surface coating but also on the cell type. Epithelial cells were more resistant to AgNPs (5–100 μg/mL) than macrophages. AgNPs decreased adherence capacity and cytotoxicity in human colon (Caco-2) cells through the generation of ROS [39] and induced ROS-mediated DNA damage along with cell cycle arrest in renal epithelial cells [40] and C3A cells [41]. Various mammalian cell lines such as macrophages (RAW 264.7, J774.1), A549, A498, HepG2, and neurons (Neuro 2A) were exposed to 43-nm AgNPs at a concentration of 2.0 mg/L for 72 h. Results were unique to each cell line. A498 and RAW 264.7 cells had the highest sensitivity, and A549 cells were the least sensitive [42]. The effects of size, surface coating, and intracellular uptake were investigated in HepG2 cells. Observations from this study indicated that both AgNPs and AgNO_3_ activate the E2-related factor 2/antioxidant response element (Nrf-2/ARE) stress response pathway, with smaller (average 10 nm diameter) AgNPs being more potent than larger AgNPs (75 nm). Interestingly, citrate-coated AgNPs resulted in higher intracellular silver concentrations compared with both polyvinyl pyrrolidone (PVP)-coated AgNPs and AgNO_3_ [43]. In support of the previous study, Gliga et al. [44] investigated the effect of various sizes of citrate-coated (10, 40 and 75 nm), PVP-coated (10 nm), and uncoated (50 nm) AgNPs in BEAS-2B cells. The results obtained from this study suggest that small AgNPs (10 nm) are cytotoxic to human lung cells; s associated with the rate of intracellular Ag release, a “Trojan horse” effect [44] and the solubility of AgNPs are other critical toxicity factors in lung epithelial cells. Ag ion toxicity is dependent on the solubility of the nanoparticles: for instance, 20 nm Ag nanospheres dissolved more rapidly than 110 nm Ag nanospheres in acidic phagolysosomes, causing more toxicity [45].

**Figure 2:**
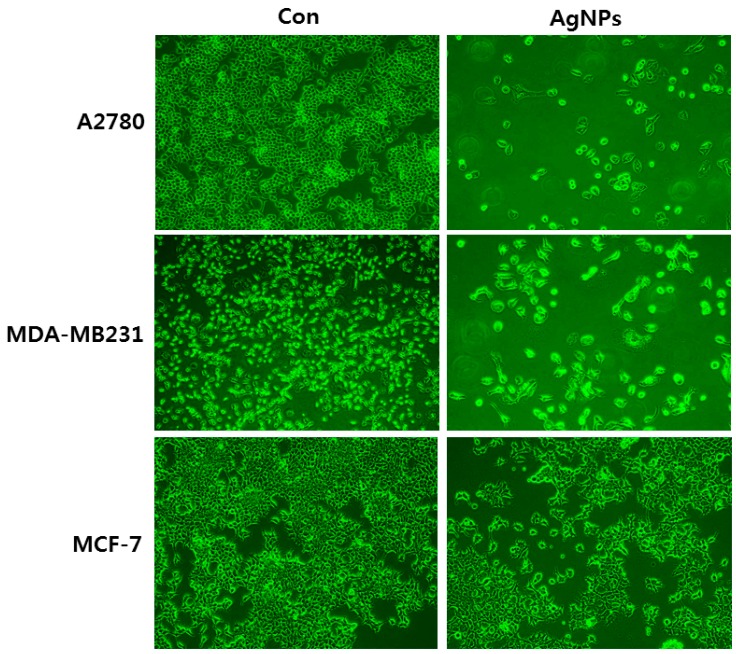
Effect of silver nanoparticles on cell morphology of human ovarian cancer cells (A2780), human breast cancer cells MDA-MB231, and MCF-7. The images were taken from the cells were treated with an average size of 40 nm for 24 h.

Exposure of HepG2 and Caco2 cells to AgNPs with an average size of 20 nm showed the dose-dependent toxic effects of DNA damage and mitochondrial injury, albeit without oxidative stress. Interestingly, among these two cell types, HepG2 were more sensitive than Caco2 cells. The authors of this study ascribe the differences in toxicity mechanisms to cell type [46]. HepG2 cells were exposed to a low concentration of two different sized AgNPs (10 and 100 nm), which exhibited increased proliferation, activation of mitogen-activated protein kinases (MAPK), and enhanced expression of c-Jun and c-Fos mRNA [47]. In other cases, the exposure of human lung epithelial cells to AgNPs causes severe cytotoxic and genotoxic effects via regulation of oxidative stress and pro-inflammatory responses [48]. Jeong et al. [49] assessed the effect of AgNPs at concentrations between 3 and 50 µg/mL under hypoxic and normoxic conditions, using a variety of cell lines including A549, normal lung epithelial cells (L132), human ovarian cancer cells (A2780), and human breast cancer cells (MCF-7 and MDA-MB 231). There was a significant difference in viability between control and AgNP-treated cancer cells, whereas there was no significant difference in the viability of untreated and AgNP-treated non-cancer L132 cells. The effect of AgNPs was more pronounced under normoxia than hypoxia conditions. Although all the cancer cells showed the same trend of decreased viability, the degree of sensitivity of cells to AgNPs varied in the order of A549, A2780, MCF-7, and MDA-MB 231. This study presents strong evidence that the responses of each cell type are different from one another [49]. While exposing all the cell types, including A2780, MCF-7, and MDA-MB 231, to AgNPs with the same average size of 40 nm at the particular concentration of 10 μg/mL, each cell type shows differential toxicity. For example, ovarian cancer cells (A2780) show more sensitivity than breast cancer cells (MDA-MB 231), which in turn were more sensitive than MCF-7 cells (Figure 2). These results indicate that each cell type has its own specific response to the external insult caused by nanomaterials. Furthermore, they suggest that the toxicity of AgNPs is influenced by the type of reducing agents used for synthesis, along with the size, shape, and surface coating.

## 3. Cellular Effects of AgNPs on Macrophages

Macrophages are large, specialized, and important immune cells that are activated in response to an infection or the accumulation of damaged or dead cells. Several studies have reported that the effect of AgNPs on macrophages varies with the concentration, incubation time, and physical and chemical features. The effect of various sizes of AgNPs (15, 30, and 55 nm) at various concentrations was evaluated using alveolar macrophages, which provide defense mechanisms against infection and play an important role in oxidative stress. After 24 h of exposure, increasing concentration of AgNPs (10–75 μg/mL) decreased viability, concomitant with a 10-fold increase in ROS levels. Furthermore, AgNPs increased the levels of cytokines and chemokines, eventually inducing an inflammatory response. Ultimately, this indicates that nanoparticle size is crucial for toxicity [15]. However, Ag-tiopronin nanoparticles with an average size of 5 nm, at high concentrations up to 200 µg/mL, have no significant effect on cytotoxicity. Interestingly, they do have a remarkable effect on interleukin (IL)-6 secretion. Murine peritoneal macrophages exhibited decreased cell viability and nitric oxide (NO) production in response to AgNPs [50,51]. U937 cells were exposed to different sizes of AgNPs (4, 20, and 70 nm) for 24 h, with the 4 nm particles having the highest toxicity, accompanied by reduction of cell viability, increased ROS generation, and greater IL-8 production when compared to the larger sizes. Smaller particles have a higher overall surface area and higher activity, and are easily transported into the cell. Although all the nanoparticles used were uniform in shape and surface coating, the size of nanoparticles is a critical factor in determining inflammatory immune response [52]. Nishanth and coworkers [53] investigated the mechanisms of the AgNP-induced inflammatory response, where they found increased IL-6 production, increased ROS concentration, nuclear translocation of NF-κB, induction of cyclooxygenase-2 (COX-2), and increased tumor necrosis factor-alpha (TNF-α) mediated by NF-κB signaling pathways. Interestingly, biologically prepared AgNPs using fibrinolytic enzyme from *Bacillus cereus* have no significant toxicity up to 100 µg/mL in the murine RAW 264.7 macrophage cell line. This study shows that bio-AgNPs are biocompatible with macrophages [54]. Similarly, chitosan-stabilized AgNPs are non-toxic to RAW264.7 cells based on a DNA fragmentation study [55].

The mechanism of toxicity of nanoparticles depends on nanoparticle properties such as surface area, size and shape, capping agent, surface charge, purity, structural distortion, and bioavailability [56]. To evaluate the effect of surface coating on toxicity, Suresh and co-workers investigated the effect of particles with uniform size and shape but with different surface coatings including poly(diallyldimethylammonium) chloride-Ag, biogenic-Ag, colloidal-Ag (uncoated), and oleate-Ag on RAW-264.7 cells. Cytotoxicity was evaluated using various properties including cell morphology, cell viability, LDH leakage, and the dissolution of silver ion concentration. The cytotoxicity of AgNPs is not merely influenced by a single characteristic, but multiple factors such as the cell type, particle aggregation, solubility, coating materials, and the surface charge [29]. Another group investigated the effect of high and low surface potentials, using tannic acid reduced (TSNPs) and sodium borohydride reduced (BSNPs) AgNPs, respectively, in RAW264.7 cells. Toxicity was evaluated by measuring changes in cellular morphology, ROS generation, metabolic activity, and the expression of various stress markers including P38 mitogen-activated protein kinases (p38) TNF-α and HSP-70. Interestingly, both AgNPs showed dose-dependent toxicity; however, TSNPs had a higher toxicity than BSNPs [57]. Pratsinis et al. [58] demonstrated the effect of different coatings by using AgNPs with well-defined sizes of 5.7 and 20.4 nm to treat murine macrophages Uncoated AgNPs had a compromised silver ion release into the cells, whereas a silica coating increased silver ion release up to a concentration of 50 mg/L. The findings from this study suggest that the release of silver ions from the surface of small nanosilver particles is significantly higher in macrophages. When the macrophages were exposed to water-dispersible AgNPs, stabilized by Ag-C σ-bonds, toxicity was observed at higher concentrations (50–500 µg/mL) and cells exhibited vesicles with an expanded volume, membranolytic action, and inflammatory responses [59].

Although many studies have claimed that AgNPs induce cytotoxicity in macrophages, Yilma et al. [60] reported the anti-inflammatory effects of silver-polyvinyl pyrrolidone (Ag-PVP) nanoparticles with sizes of 10, 20, and 80 nm in mouse macrophages infected with live *Chlamydia trachomatis*, a very common sexually transmissible infection. These findings suggest that Ag-PVP nanoparticles (10 nm) selectively inhibit prototypic cytokines TNF-α and IL-6, as elicited from *C. trachomatis* and a broad spectrum of other cytokines and chemokines produced by infected macrophages. Action appears to occur through alteration of a variety of receptor proteins and inflammatory signaling pathways by downregulating their messenger ribonucleic acid (mRNA). Similarly, biologically synthesized AgNPs exhibit anti-inflammatory activity against hydrogen peroxide-induced nitric oxide as well as superoxide anions in rat peritoneal macrophages [61]. Recently, Nguyen et al. [62] studied the effect of OECD (Organization for Economic Co-operation and Development) representative AgNPs, NM300K, on the mouse macrophage line J774A.1 using several parameters. When the cells were exposed to various concentrations up to 250 µg/mL for 24 h, there was a dose-dependent decrease in cell viability. At high doses, NM300K altered cell shape and induced the formation of vacuolar structures, increased levels of cytokines, and increased ROS production, leading to oxidative DNA damage and apoptosis. The results from these studies suggest that Ag^+^ released from NPs by dissolution could be a primary contributor to toxicity.

## 4. Cellular Effects of AgNPs on Endothelial Cells

Angiogenesis is the generation of new blood vessels [63]. Angiogenesis plays a significant role in cancer, diabetic retinopathy, and rheumatoid arthritis, among other diseases. Therefore, it is necessary to study the effect of AgNPs in various angiogenic-related diseases using various culture systems. Kalishwaralal et al. [64] investigated the effect of biologically synthesized 50 nm spherical AgNPs at a concentration of 500 nM in bovine retinal endothelial cells (BRECs) in the presence and absence of vascular endothelial growth factor (VEGF). They found that AgNPs can inhibit cell survival, VEGF-induced cell viability, cell proliferation, and migration through the activation of caspase-3 and suppression of Akt phosphorylation [64,65]. The possible inhibition of phosphorylation of Akt was specific targeting of Thr308 and Ser473 residues of Akt by AgNPs. Molecules can have dual functions depending on their concentration, with concentration effecting toxicity and biocompatibility. Taking this into account, Rosas-Hernández et al. [63] investigated the dose-dependent effects of chemically prepared AgNPs with an average size of 45 nm in coronary endothelial cells, specifically studying biological effects such as cell proliferation and nitric oxide (NO) production. They found that AgNPs had a dual effect with regards to cell proliferation, whereby proliferation was inhibited at low concentrations of NPs and stimulated at high concentrations [63].

High vascular permeability is one of the major problems of angiogenic-related diseases including diabetic retinopathy, which is regulated by various growth factors and cytokines [16]. To develop a new therapeutic strategy, AgNPs were used to inhibit cytokines that enhance vascular permeability. The results from Sheikpranbabu and co-workers showed that AgNPs could block the cytokine-induced permeability in porcine retinal endothelial cells (PRECs) through modulation of Src kinase phosphorylation. In another approach, we demonstrated that AgNPs can inhibit advanced glycation end-product (AGE)-induced endothelial cell permeability via upregulation of tight junction proteins such as occludin and ZO-1. AgNPs are able to cross the blood–brain barrier through transcytosis and were observed to accumulate in the rat brain microvessel vascular endothelial cells [23]. Trickler et al. [19] studied blood–brain barrier inflammation and permeability in primary rat brain microvessel endothelial cells using 25, 40, or 80 nm AgNPs. This study concluded that smaller sizes induced significant effects at all concentrations and time points. A study from rat brain endothelial cell culture suggests that AgNP toxicity depends on particle size, surface area, dose, and exposure time [66].

**Figure 3:**
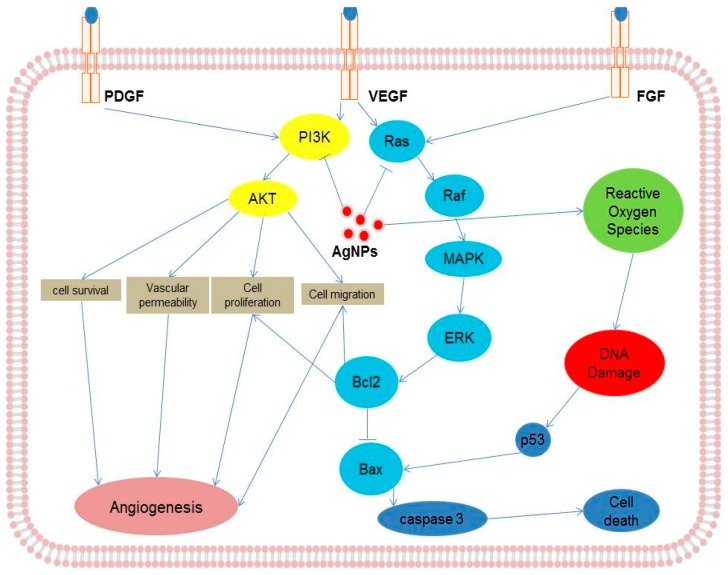
Possible signaling pathways of inhibition by silver nanoparticles on various angiogenic processes induced by growth factors in endothelial cells. PDGF, platelet-derived growth factor; VEGF, vascular endothelial growth factor; FGF, fibroblast growth factor; RAS, Ras is a membrane-associated guanine nucleotide-binding protein; RAF, RAF kinases are a family of three serine/threonine-specific protein kinases; MAPK, mitogen-activated protein kinases; ERK, extracellular-signal-regulated kinases; PI3K, phosphatidylinositide 3-kinases; AKT, also known as protein kinase B (PKB); Bcl-2, B-cell lymphoma 2; Bax, BCL2 associated X; p53, tumor suppressor p53.

Coating is an important aspect in determining nanoparticle biocompatibility or toxicity. A study was performed by Kang et al. to investigate the effect of polyvinylpyrrolidone (PVP)-coated AgNPs (average size 2.3 nm) on angiogenesis using both an in vivo and an in vitro model. The results indicated that coated AgNPs induce angiogenesis through production of ROS and angiogenic factors, and they activate focal adhesion kinase (FAK), Akt, ERK1/2, and p38, which are all involved in VEGF receptor (VEGFR)-mediated cell survival signaling [67,68]. VEGF is known to promotes the activation of VEGF receptor-2 (KDR/Flk-1), which in turn phosphorylates FAK, PI3K/Akt, extracellular-signal-regulated kinases (ERK1/2), mitogen-activated protein kinases (p38), and endothelial NOS (eNOS), proteins that regulate survival, migration, proliferation, and vasodilatation [67,68]. Previously, various studies demonstrated that AgNPs could block the VEGF-induced signaling pathway by in activation or dephosphorylation of respective candidates in endothelial cells [64,65,66,67,68,69]. Mukherjee et al. [69] demonstrated the biocompatibility of biologically synthesized AgNPs in human umbilical vein endothelial cells (HUVEC). They found that cells incubated with biosynthesized AgNPs showed no toxicity response at a concentration of 30 µM compared to chemically synthesized AgNPs. Conversely, AgNPs inhibited proliferation, damaged the cell membrane, induced apoptosis, and upregulated inflammatory cytokines, adhesion molecules, and chemokines in HUVECs through the activation of NF-κB pathways [70]. Endothelial cells are known to induce angiogenesis in the presence of various growth factors such as VEGF, platelet-derived growth factor (PDGF), and fibroblast growth factor (FGF). The possible inhibitory effects of AgNPs on various receptor-mediated signaling pathways in endothelial cells are shown in Figure 3.

**Figure 4:**
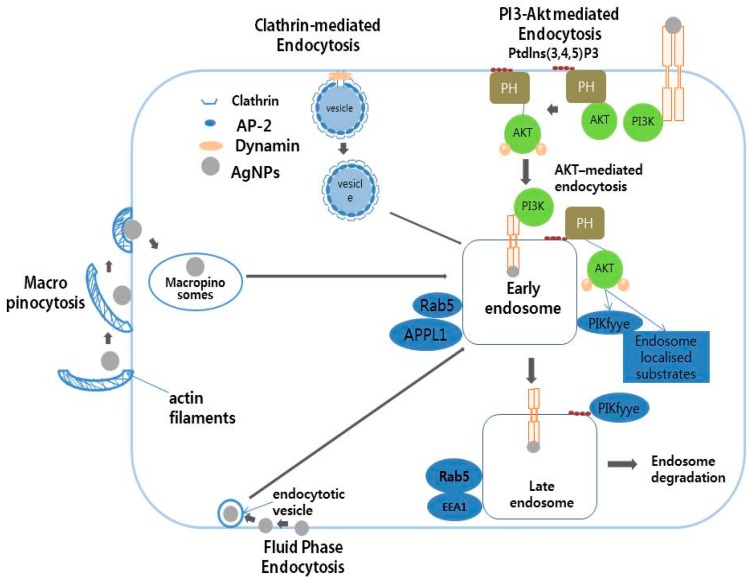
The schematic diagram represents the possible cellular uptake of silver nanoparticles by active and passive processes in eukaryotic cells. AP-2, Adaptor complex; PH domain, pleckstrin homology domain; PKB/*Akt*, Protein *kinase* B or *Akt*; PI3K, phosphatidylinositide 3-kinases; Rab5, Ras related protein; APPL1, a4 precursor protein like 1; EEA1, early endosome-associated protein.

Endocytosis is an important mechanism for the intracellular trafficking of nanoparticles (NPs). NPs have been shown to enter into the cells through early endosomes, late endosomes, and lysosomes, with endocytosis being a major mechanism underlying the cellular uptake of AgNPs [44]. AgNPs and their protein coronas are dependent on several factors including size, concentration, and surface functionalization. AgNP protein coronas influence several biological responses including oxidative stress, inflammation, and cytotoxicity as well as cellular bio-physicochemical mechanisms such as endocytosis, biotransformation, and bio-distribution [71]. A protein corona study was performed using citrate-suspended 20 nm AgNPs with various proteins including human serum albumin (HSA), bovine serum albumin (BSA), and high-density lipoprotein (HDL). The results obtained from this study evaluated the activity, bio-distribution, cellular uptake, clearance, and toxicity in the presence of scavenger receptor BI (SR-BI) in rat aortic endothelial cells. Thirty-five-nanometer AgNPs exposed to human microvascular endothelial cells induced cytotoxicity by increasing leakage of lactate dehydrogenase and ROS generation, and increased genotoxicity by DNA damage [72]. Cellular uptake was influenced by the physical and chemical properties of the AgNPs, which enter cells using different endocytosis mechanisms such as clathrin-mediated endocytosis, PI3/Akt-mediated endocytosis, macropinocytosis, and fluid phase endocytosis (Figure 4).

## 5. Cellular Effects of AgNPs on Keratinocytes and Fibroblasts

To evaluate the potential toxicity of AgNPs on skin tissue, they were introduced to an immortalized human keratinocyte cell line (HaCaT), recognized as a suitable model for assessing the toxicological potential of nanomaterials that can cause skin damage in vitro [73]. Short exposure of the human keratinocyte cell line HaCaT to AgNPs resulted in a long-lasting anti-proliferative effect, independent of cell penetration and ROS production [74]. The wounds were treated with either AgNPs or a silver sulfadiazine control; the results confirmed that AgNPs increase the rate of wound closure by enhanced proliferation and migration of keratinocytes [75].

In the case of fibroblasts, AgNPs induced differentiation into myofibroblasts. A study was performed to characterize the resistance of CCL-153 and RTgill-W1 cells when exposed to two different sizes of AgNPs (10 or 100 nm). Interestingly, 100 nm AgNPs elicited overall lower resistance values and 10 nm AgNPs elicited almost negligible resistance with stronger cytotoxicity. AgNPs with a smaller size have the ability to enter into cells more easily, thereby decreasing the cell attachment and thus lowering resistance values [76]. The effect of a heat-treated polyvinyl alcohol (PVA) nanofibrous matrix containing Ag ions and/or nanoparticles on normal human epidermal keratinocytes (NHEK) and fibroblasts (NHEF) has also been studied. The PVA nanofibrous matrix containing Ag induced increased attachment and spreading in early stage culture when compared to the PVA nanofibers without Ag. Of these two cell lines, NHEF cells appeared to be more sensitive to Ag ions or particles than NHEK cells [77]. Experiments using primary NHEK cells treated with AgNPs showed a concentration-dependent toxic effect on cell viability, proliferation, migration, and metabolic activity [78]. However, schizophyllan (SPG)-mediated synthesis of AgNPs with an average size of 6 nm were non-toxic to a mouse fibroblast line (NIH-3T3) and HaCaTs [79].

Cytoxicity of 2-acrylamido-2-methylpropane sulfonic acid sodium salt with AgNPs was evaluated to assess the efficacy of novel commercially available silver composites in two different cell model systems, HaCaT primary keratinocytes (HEK) and normal human fibroblasts (NHF), at several time points. The commercially available Acticoat™ and Flamazine™ creams were toxic depending on exposure time and cell type. A novel silver hydrogel and PolyMem Silver^®^ had low cytotoxicity to all tested cell lines at every time interval. It is of note that the results from HaCaTs and HEKs were significantly different, emphasizing the importance of testing in multiple cell types under different conditions when developing any drug molecule [80]. Recently, the effects of silver ions and AgNPs were investigated in two different cell lines, human dermal fibroblasts (NHDF) and NHEKs. The findings from this study showed that Ag ions were significantly more toxic than AgNPs to both cell types. Interestingly, non-cytotoxic concentrations of AgNPs and Ag-I did not induce DNA damage and did not affect inflammatory markers. The authors concluded that AgNPs are more suitable for application as a topical agent for wound healing.

Nanosilver toxicity depends on the release of Ag^+^. The toxicity of ions in cellular systems depends on several parameters including surface coating, coexisting molecules, especially thiol-containing compounds, lighting conditions, and the interaction of nanosilver with nucleic acids, lipid molecules, and proteins [81]. Nanosilver interacts with cellular membranes and causes membrane damage, including increasing membrane permeability. The damage caused to the cell membrane is due to intracellular calcium overload, and further causes ROS overproduction and mitochondrial membrane potential variation [82]. One of the vital aspects of Ag^+^ ion toxicity is oxidative stress and the induction of imbalances between pro-oxidant and anti-oxidant molecules in the cellular systems. Ag^+^ causes toxicity through interaction with proteins and amino acids [81,83].

## 6. Cellular Effects of AgNPs on Neuronal Cells

The neurotoxicity of AgNPs with an average size of 15 nm at a concentration of 10 µg/mL for 24 h was investigated using the dopaminergic neuronal cell line PC12. From the results of this study it can be inferred that silver is involved not only in oxidative stress but also in alteration of enzymatic functions, which play important roles in dopamine depletion [84]. The neurotoxic effects of AgNPs, AgAc (silver acetate), and the 12 kDa filtered sub-nano AgNPs fraction was assessed in PC12 cells. Both AgNPs and the 12 kDa filtered fraction produced similar effects, whereas AgAc was significantly more potent. Interestingly, AgNPs did not induce necrosis. On the other hand, death receptor-mediated apoptosis was observed in cells treated with AgNPs and the 12 kDa filtered fraction [85]. AgNPs significantly induce cellular toxicity in cerebellum granule cells (CGCs) in a dose-dependent manner without damaging the cell membrane, through activation of caspase-3, oxidative stress, depletion of anti-oxidant molecules, and reducing intracellular calcium levels [86]. When primary rat cortical cell cultures were exposed to various concentrations of AgNPs from 1 to 50 µg/mL, neurite outgrowth was inhibited and the cell viability of premature neurons and glial cells was reduced via mitochondrial dysfunction and loss of cytoskeleton proteins including β-tubulin and filamentous actin (F-actin). Furthermore, AgNPs significantly reduced the abundance of the presynaptic vesicle protein synaptophysin, and the postsynaptic receptor density protein PSD-95 [25].

**Figure 5:**
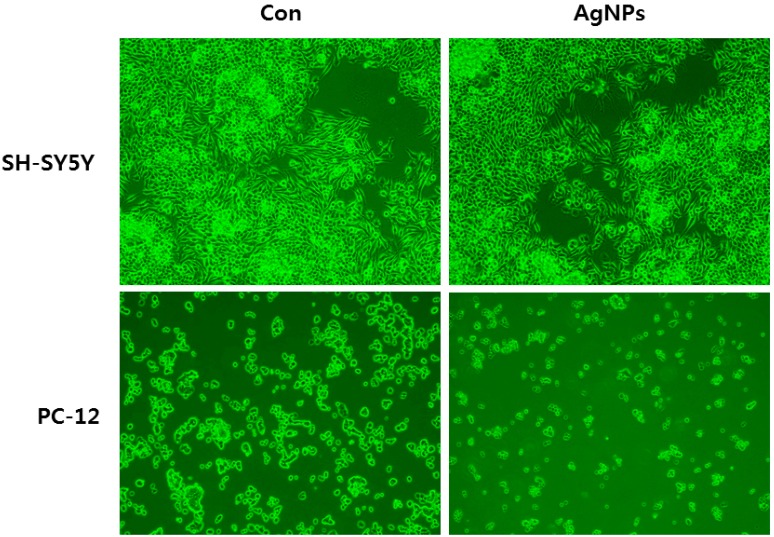
Effect of silver nanoparticles on cell morphology of human neuroblastoma SH-SY5Y cells and pheochromocytoma (PC-12) cells. The images were taken from cells with an average size of 40 nm treated for 24 h.

Biologically synthesized AgNPs with an average size of 30 nm induce neuronal differentiation in neuroblastoma cancer cells (SH-SY5Y cells) through modulation of ROS, phosphatases, and kinase signaling pathways. Interestingly, Dayem et al. [26] observed that cells treated with AgNPs had significantly altered cell morphology and neurite length caused by enhanced expression of neuronal differentiation markers such as Map-2, β-tubulin III, synaptophysin, neurogenin-1, Gap-43, and Drd-2 [26]. Similarly, another group from Israel found that substrates coated with AgNPs, serving as favorable anchoring sites, significantly enhanced neurite outgrowth [87]. The effects of size and coating of AgNPs of 10 and 75 nm were studied in N27 rat dopaminergic neurons coated with either PVP or citrate. Both coated AgNPs influenced caspase-3 activity and glutathione content. Of the two different coated AgNPs, 75 nm PVP coated was significantly active and enhanced global gene expression, especially oxidative stress pathways, and 10 nm PVP coated AgNPs influenced the NRF2 pathway [88]. PC-12 cells showed more sensitivity than SH-SY5Y when treated with 40 nm AgNPs (Figure 5). The size and surface coating of nanoparticles play crucial roles in toxicity and the cellular response of cells against nanoparticles.

Functionalization plays an important role in nanoparticle-induced toxicity. For instance, Kennedy et al. [89] investigated AgNPs functionalized with three different monosaccharides such as d-glucose, d-mannose, d-galactose, citrate, and ethylene glycol in Neuro-2A and HepG2 cells. The findings from this study suggest that toxicity decreased when AgNPs were coated with galactose and mannose, compared to particles that were coated with glucose, ethylene glycol, or citrate. Toxicity is mainly correlated with intracellular oxidative stress, not uptake efficiency of AgNPs by cells [89]. To overcome the toxicity induced by AgNPs, Ma et al. [90] proposed protective measures by simultaneously adding selenium to murine hippocampal neuronal HT22 cells [90]. To evaluate the protective effect against AgNP-induced toxicity, the cell viability, ROS production, caspase-3 activity, mitochondrial oxygen consumption, and mitochondrial membrane potential were measured following treatment. As they expected, selenium protected the cells from the adverse effects of AgNPs. Mytych et al. [91] studied the prolonged effects of AgNPs in HT22s by treating the cells with a low concentration of AgNPs (5 μg/mL) for 48 h, and evaluated the cytophysiological effects 96 h following AgNP removal. Surprisingly, no toxicity was observed; conversely, the AgNPs modulated the HT22 proliferation and induced oxidative stress and 53BP1 recruitment. For the first time, they showed that AgNPs stimulate changes in DNA methylation.

AgNPs are known to impair neuronal cell functions, for instance, in rat cerebellum granule cells. Exposure to AgNPs induces neurotoxicity and eventually leads to apoptosis through oxidative stress [86]. Astrocytes are the predominant cell type in the brain, with a vital role in the growth and maintenance of capillaries and other brain cells by regulating gliogenesis and synaptogenesis. They are also involved in the detoxification of xenobiotics and ROS, modulation of BBB permeability, and act as mediators of neurotoxicity [92,93,94]. Primary rat astrocytes exposed to PVP-coated AgNPs for up to 24 h at a concentration of 10 µg/mL accumulated AgNPs in a time- and concentration-dependent manner. The accumulation of AgNPs had no effect on cell viability or the level of glutathione, which indicates that PVP coating might protect against silver ion-induced toxicity [95].

## 7. Cellular Effects of AgNPs in Stem Cells

Toxicity of AgNPs in stem cells was first observed in murine spermatogonial stem cells using cell morphology analysis, proliferation, and cytotoxicity assays. There was no significant difference in morphology between control and AgNP treated groups. However, cell viability, LDH leakage, and apoptosis were significantly affected in the AgNP-treated groups [96].The results suggest that both types of AgNP used reduced cell viability and increased apoptosis through upregulation of p53 and Rad51 and the phosphorylation of H2AX. Another study from Greulich et al. [97] investigated the biocompatibility of 100 nm AgNPs in human mesenchymal stem cells (hMSCs). The findings from this study showed a dose dependent effect on cytotoxicity. The cytokine level of IL-8 was significantly higher than IL-6 and VEGF at concentrations of 5 µg/mL and above. Light microscopy revealed that AgNPs were mainly located in the perinuclear region. Fluorescence microscopy showed that the AgNPs were contained mainly within endolysosomal structures, not in the cell nucleus, endoplasmic reticulum, or Golgi complex. This implicates clathrin-dependent endocytosis and micropinocytosis as the main entry routes of AgNPs into human mesenchymal stem cells (hMSCs).

The distribution of AgNPs in the cells and AgNP-induced DNA damage, cell death, and functional impairment in hMSCs were analyzed after different exposure times of 1, 3, and 24 h. Distribution analysis showed that AgNPs were mainly located in the cytoplasm and the nucleus. Furthermore significant increases in IL-6, IL-8, and VEGF were observed. The exposure of hMSCs to higher concentrations induced cytotoxic and genotoxic effects. Interestingly the migration ability of hMSCs was not impaired at sub-toxic concentrations [98]. Conversely, Samberg et al. [99] performed experiments on human adipose-derived stem cells (hASCs) in both undifferentiated and differentiated states, treating them with 10 or 20 nm AgNPs at various concentrations from 0.1 to 100 µg/mL and no significant cytotoxicity was observed. Similarly, in hMSCs and osteoblasts (OBs) cytotoxicity and the inhibition of proliferation were time and dose dependent. Interestingly, there was no significant effect on cell differentiation. These findings suggest that AgNPs could be used as biocompatible agents within a particular dosage window [100]. hMSCs exposed to different concentrations of AgNPs exhibited no significant changes in alkaline phosphatase activity, osteocalcin gene expression, osteopontin expression, and mineralization [101]. Similarly, AgNPs had no effect on osteogenic differentiation when human urine-derived stem cells were incubated with non-cytotoxic concentrations [102].

**Figure 6:**
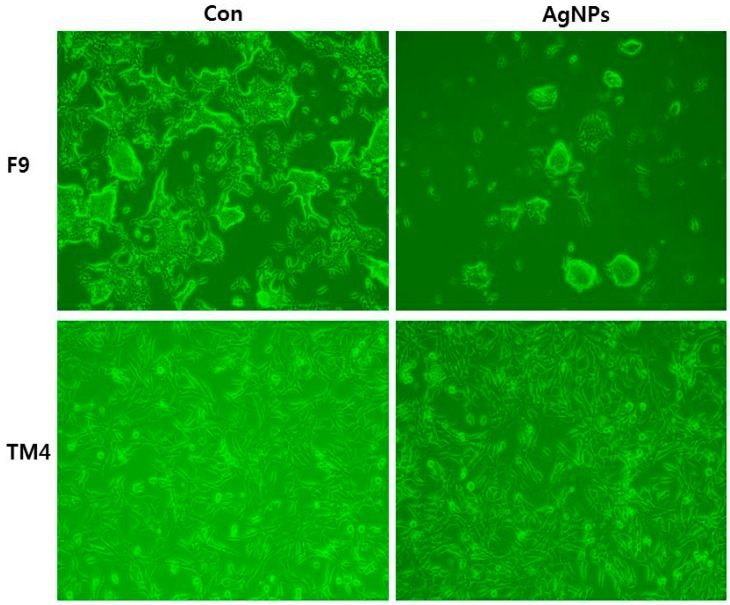
Effect of silver nanoparticles on cell morphology of teratocarcinoma stem cell line F9 and Sertoli (TM4) cells treated with 40 nm AgNPs. The images were taken from cells with an average size of 40 nm treated for 24 h.

Conversely, the report from Söderstjerna and co-workers suggests that AgNPs have significant effect on sphere size and morphology in human embryonic neural precursor cells [103]. Similarly, Zhang et al. [104] reported that AgNP had a significant effect on male somatic Leydig (TM3) and Sertoli (TM4) cells and spermatogonial stem cells (SSCs), which varied with size and cell type. AgNPs induced apoptosis through mitochondrial damage and activation of various signaling pathways including those involving p53, p38, and pErk1/2. AgNPs also induced accumulation of autosomes and autolysosomes. In the case of embryonic neural stem cells (NSCs) exposed to various concentrations of AgNPs for 24 h, neurotoxicity was observed by decreased cell viability, enhanced leakage of LDH and ROS, upregulation of Bax protein, and increased apoptosis [105]. The same neural stem cells exposed to low concentrations of AgNPs (1 µg/mL) induced formation of f-actin filaments, resulting in disruption of actin dynamics, reduction in neurite extension, and branching of cells [106]. AgNPs therefore have a strong negative effect on neurogenesis. Sertoli (TM4) cells had a lower sensitivity than the teratocarcinoma stem cell line F9 when treated with 40 nm AgNPs (Figure 6).

## 8. Mechanism of Action of AgNPs in Various Cellular Systems

The working principles and mechanism of action of AgNPs are vital and different in each and every cell type. Induction of cytotoxicity and genotoxicity by AgNPs depends on many factors such as size, shape, surface charge, surface coating, solubility, concentration, media, surface functionalization, distribution into the cells, mode of entry, and cell type. Recently, Duran and co-workers extensively reviewed the toxicity of AgNPs based on protein coronas [71]. The biological activities of AgNPs depend on proteins present in the cell culture media: the presence of a protein corona contributes to toxicity and facilitates interactions between AgNPs and cells to induce or mitigate toxicity [71]. After introduction of AgNPs to cells, the initial events are entry into and distribution within the cells, a critical event for determining toxicity. AgNPs penetrated into the cells through several cellular compartments including endosomes, lysosomes, and mitochondria [13]. The probable mechanisms of NPs uptake by cells include pinocytosis, caveolae- and clathrin-dependent mediated endocytosis, and phagocytosis [13].

Once AgNPs enter into cells, cell fate is determined by several factors including the efficacy of anti-oxidative defense, the efficiency of DNA repair systems, apoptotic propensity, and cellular signaling mechanisms [107]. The probable mechanisms of AgNP-induced cytotoxicity and genotoxicity are ROS-mediated apoptosis and necrosis [12,13,15,52] depending on the size and concentration. For instance, the entry of AgNPs into an endothelial cell monolayer through the endolysosomal pathway affects cell morphology and the function of BBB [108], subsequently affecting various biological molecules such as DNA, proteins, and lipids. Furthermore, interaction of AgNPs with biomolecules could induce many cellular and biochemical changes including oxidative stress, conformational alteration, increased membrane permeability, mutations, activation of signaling pathways, ionic exchange disorder, enzyme failure, and new protein epitope exposure [109]. Several in vitro models suggest that ROS-mediated toxicity is more pronounced and causes cellular and biochemical alteration in the cells [110]. The most probable mechanism of AgNP-induced toxicity is oxidative stress.

ROS, which are highly reactive molecules including the superoxide radical (O^2−^) and H_2_O_2_, are the main cause of oxidative stress. ROS are a double-edged sword, as under normal physiological conditions a moderate level of ROS is essential to maintain normal physiological processes. However, excessive ROS, exceeding the capacity of the cellular antioxidant defense system, leads to cellular damage targeting proteins, lipids, and DNA [24,108,111,112,113]. Mitochondria are the main intracellular source of ROS. Several studies have reported that AgNPs can accumulate outside the mitochondria and cause mitochondrial dysfunction, also disturbing the respiratory chain function and resulting in ROS generation and oxidative stress [13]. ROS also disrupt the mitochondrial respiratory chain and ATP synthesis, eventually leading to DNA damage [10,11,13]. ROS overproduction can lead to the opening of the mitochondrial permeability transition pore (MPTP), resulting in apoptosis in mammalian cells. AgNPs induce apoptosis through release of cytochrome *c* into the cytosol and translocation of Bax to the mitochondria, and also cause cell cycle arrest in the G1 and S phases [114,115]. The main result of AgNP toxicity is direct and oxidative DNA damage, ultimately causing apoptosis [81].

Inflammation is responsible for toxicity and promotes cell death in cells through ROS and complementary proteins, as well as via receptor-induced apoptosis/necrosis [116]. Inflammatory and immune responses are regulated by multiple signaling pathways such as NF-κB and mitogen-activated protein kinase including extracellular-signal-regulated kinases (Erk1/2), mitogen-activated protein kinases (p38), cAMP response element-binding protein (CREB), cMyc, and c-Jun N-terminal kinase (JNK) pathways [117]. Normal functioning of cells depends on an intact and functioning immune system, and controlled inflammation is necessary to maintain homeostasis [118]. Several in vitro studies have reported that AgNPs induce an inflammatory response in cells. The release of inflammatory cytokines is directly linked with ROS, which seem to be the main candidate for AgNP-induced inflammation. Inflammatory factors are able to cause DNA damage and may prevent DNA repair and promote aberrant methylation patterns leading to changed gene expression profiles [119,120]. AgNP-induced cytokines include IL-1, IL-6, and TNF-α as well as IL-1β, which is an indirect marker of inflammasome activation [118]. Orłowski et al. [121] found that AgNPs induce activation of caspase-1 in a monocyte cell line. Active caspase-1 is involved in formation of the inflammasome complex, which is a major player in pyroptosis [122,123]. The characteristic features of pyroptosis include rapid formation of plasma membrane pores and DNA damage, resulting in cell lysis and release of inflammatory intracellular contents.

Oxidative DNA damage is the final cellular event of apoptosis, which is pronounced in many diseases including cancer, neurodegenerative disorders, and cardiovascular disease, and in aging due to exogenous insult of the overdosage of toxic materials or nanomaterials. AgNP-mediated excessive ROS induces DNA damage through oxidation of DNA bases, apurinic/apyrimidinic (AP) sites, single strand breaks, and base lesions [124]. AgNPs induce DNA damage in various types of mammalian cells via chromosomal aberrations, DNA strand breaks, oxidative DNA damage, and mutations [125,126]. DNA damage can also be caused by AgNP-mediated gene upregulation of, for example, DNA-damage-inducible transcript 3 (DDIT3), caspase 1, apoptosis-related cysteine peptidase (CASP1), and growth arrest and DNA-damage-inducible protein (GADD45A) alpha. They can also cause chromosome instability and inhibit mitosis [127]. When OH^•^ reacts with all components of DNA causing DNA single strand breakage, an 8-hydroxyl-2′-deoxyguanosine (8-OHdG) DNA adduct is formed, which is a biomarker of OH^•^-mediated DNA lesions [128].

## 9. Activation of Signaling Molecules in Response to AgNPs in Various Cell Culture Systems

Signal-transduction cascades mediate communication between cells, creating a molecular circuit to generate responses such as changes in enzyme activity, gene expression, ion-channel activity, or phosphorylation or dephosphorylation by kinases and phosphatases, respectively. Cell proliferation is mainly regulated by mitogen-activated protein kinases (MAPKs) in many physiological activities, and high doses of AgNPs are able to upregulate MAPKs in mammalian cells [129,130]. MAPKs are a group of serine/threonine proteins including extracellular signal-regulated kinase (ERK), p38MAPK, and JNK, which modulate many cellular responses against AgNPs [131]. AgNPs stimulated the expression of p38pp, c-Jun, and c-Fos, suggesting that the p38 MAPK mediated pathway is involved in cell protection under AgNP-induced stress [47]. Activation of the PI3K/Akt pathway plays a crucial role in the survival of variety of cells under growth factor stimulated conditions. AgNPs can inhibit phosphorylation of the p85 subunit of PI3K: the activity of PI3K seemed to be the primary target of AgNPs, and inhibition of Akt is a consequence of this effect [65]. Activation of several MAPKs plays an important role in AgNP-induced toxicity in various cell lines. MAPKs are involved in AgNP-activated p38 and pErk1/2 signaling pathways in spermatogonial stem cells [129]. AgNPs induce apoptosis in spermatogonial stem cells through increased levels of ROS; mitochondrial dysfunction; upregulation of p53 expression; pErk1/2; and accumulation of autophagosomes, autolysosomes, and autophagolysosomes [104].

Recently, endoplasmic reticulum (ER) stress-induced apoptosis caused by AgNPs has attracted much research interest. The ER stress response constitutes a cellular process that is triggered by a variety of conditions that disturb the folding of proteins in the ER [132]. It is known that AgNPs induce apoptosis via modulation of ER stress [133]. AgNPs controlling up- and downregulation of various genes that are involved in ER stress and also phosphorylation of RNA-dependent protein kinase-like ER kinase (PERK), inositol-requiring protein 1 (IRE1), splicing of ER stress-specific X-box transcription factor-1, cleavage of activating transcription factor 6 (ATF6), and upregulation of glucose-regulated protein-78 and CCAAT/enhancer-binding protein-homologous protein (CHOP/GADD153). In order to protect the cells against nanoparticle-mediated toxicity, the ER rapidly responds with the unfolded protein response (UPR), an important cellular self-protection mechanism [134]. The ER stress system contains three dominant stress sensors, IRE1, PERK, and ATF-6 [135]. For instance, the activation of the ER stress signaling pathway was observed by upregulated expression of xbp-1s, chop/DDIT3, TRIB3, ADM2, BIP, Caspase-12, ASNS, and HERP at either the mRNA and/or protein levels in 16HBE cells. Conversely, there was no significant upregulation in HUVECs or HepG2 cells [135]. Simard et al. [136] attributed ER stress-mediated apoptosis to low and high concentrations of 15 nm AgNPs. Interestingly, at lower concentrations, exposure of WT human monocytic THP-1 cells to AgNPs had no significant effect on cell death, whereas higher concentrations resulted in an atypical ER stress response associated with ATF-6 degradation and pyroptotic cell death through NLRP-3 inflammasome activation [136]. Accumulating evidence suggests that AgNP-mediated ER stress is responsible for cellular dysfunction and activation of the cell death pathway. We summarize the cellular effects of AgNPs in various cellular systems based on exposure doses, concentration, size of particles, type cell line used, and major outcome of each study in Table 1 [24,38,64,72,105,137,138,139,140,141,142,143,144,145,146,147,148].

## 10. Conclusions and Future Perspectives

Nanotechnology is a rapidly growing field. In particular, AgNPs have applications in many industries including biomedicine, pharmaceuticals, biotechnology, nanomedicine, and instrumentation as antibacterial agents, drug delivery agents, biosensors, and environmental sensors due to their unique properties. AgNPs offer significant specificity with enhanced bioavailability compared to existing conventional therapeutic agents. To understand the toxicity and biocompatibility of AgNPs, several cell lines have been used as in vitro model systems. In this review, to summarize the differential responses of several types of cell lines against AgNPs, we have chosen important cell types involved in various diseases. Several studies concluded that the cellular response of each cell type depends on: physical and chemical properties; concentration; incubation time; presence of serum, and hence protein coronas, which influence cellular uptake; ion release; bio-distribution; biological activity; toxicity; and biocompatibility. Secondarily important aspects of the cellular response include agglomeration in cell medium, intracellular localization, and Ag release. The common mechanisms of AgNP toxicity, ROS-mediated mitochondrial dysfunction, DNA damage, and apoptosis, were observed in most of the cell lines. Inflammatory responses and signaling molecules involved in AgNP-exposed cells were unique for every cell type.

Although large numbers of studies have addressed nano-toxicity or cellular behavior, data gaps remain due to sample formulation, treatment, design, concentration, model type, and several other factors. Taking all these parameters into consideration, understanding how AgNPs interact with particular cells and stimulate signaling cascades and the reasons for the wide variety of responses in different cell lines is a complex task. Any toxicology study of a new nanomaterial requires an in vitro biocompatibility assessment using different cell types in various cell culture media formulations, and nanomaterial suspensions with the same physical and chemical properties simultaneously evaluated with the cooperation of many laboratories. Due to the complexity of AgNP molecular mechanisms, comprehensive computational modeling approaches will be needed to understand not only the mechanisms involved in corona formation, cellular mechanics, evolution, and dynamics, but also the dynamics of cellular proteins.

Therefore, future work should be carefully directed to avoid differential cellular responses and false toxicity interpretations. Well-characterized particles that have standardized size, shape, coating, functionalization, and ionic toxicity should be used. In particular, the genotype of the cell lines used should be taken into account and standardized where possible to exclude the influence of physical and chemical characters. Another concern for future work is to standardize conditions and experimental procedures; therefore, it is necessary to conduct interlaboratory studies and compare data from each laboratory. Evidently, obtaining a complete understanding of the biological function of AgNPs is not an easy task, but it is necessary to develop a common methodology for the development of safer nanomaterials for therapeutic applications. Furthermore, the deep understanding of mechanisms of action of AgNPs would help in the development of safe nanomaterials for nanotechnology-based consumer products without harmful side effects.

## Figures and Tables

**Table 1:** The effect of silver nanoparticles (AgNPs) on various cells lines by various concentration, doses, and sizes.

Serial Number	Exposure Doses of AgNPs	Exposure Time	Size (nm)	Type of Cell Lines Used	Major Outcomes	Reference
1	0.01 µg Ag/mL	24 h	20, 50, 75	Human pulmonary epithelial cell line 16HBE14	Dose and process of uptake	[137]
2	c0/4 and higher, 2.25 × 10^9^–1.35 × 10^10^ Wrs/mL, 9 × 10^15^–1.01 × 10^16^ nm²/mL, 3.68–3.83 mg/mL	24 h	30, 60–100	Human alveolar epithelial cells (A549)	Spherical particles had no effect than silver wires	[38]
3	30 and 278 ng/cm^2^	4 and 24 h	20	Human alveolar epithelial cells (A549)	Cells were only sensitive to high Ag-ion concentrations	[138]
4	20 and 100 µg/mL	48 h	10, 20, 75 and 110	T84 cells (ATCC CCL-248™), a human colorectal carcinoma cell line	Small AgNPs have significant effects on intestinal permeability	[139]
5	50 mg/L	24 h	61.2 ± 33.9	Porcine kidney (Pk15) cells	AgNPs had only insignificant toxicity at concentrations lower than 25 mg/L, whereas Ag+ exhibited a significant decrease in cell viability at higher concentration	[140]
6	2–6 μM	1–3 weeks	20–60	Human HCE-T corneal epithelial cells	Mammalian cell toxicity was observed at high (8–12 μM silver ion) silver levels in serum-free culture	[141]
7	2–6 μM	1–3 weeks	20–60	RAW264.7 macrophages	Low cell pro-inflammatory cytokine activation was observed	[141]
8	0.31 to 10 g/mL	48 h	10	Human tongue squamous carcinoma SCC-25	Reduced proliferation and viability	[142]
9	20 μg/mL	24 h	70	Alveolar epithelial cells, macrophages, and dendritic cells	Adverse effects were also only found at high silver concentrations	[143]
10	1.0 and 2.5 μg/mL	72 h	35	Human microvascular endothelial cells	Loss of membrane integrity at higher concentrations	[72]
11	500 nM	24 h	50	Bovine retinal endothelial cells	Enhanced apoptosis	[64]
12	500 nM	24 h	50	Dalton’s lymphoma ascites	Anti-tumor activity	[144]
13	2.0 and 4.0 mg/L	24 h	10 and 100	HepG2 cells	Non-cytotoxic doses induced p38 MAPK pathway activation and led to the promotion of HepG2 cell proliferation	[47]
14	7.74 mg/L	24–72 h	65–69	HaCaT cells,	HaCaT cells were found to be resistant	[145]
15	1.16 mg/L	24–72 h	65–69	HeLa cells	HeLa cells were found to be more sensitive	[145]
16	1–20 μg/mL	24 h	23	Embryonic neural stem cells	Ag-NPs-induced neurotoxicity	[104]
17	10–20 μg/mL	24 h	20 and 40	Primary mixed neural cell cultures	Strong effects of SNP associated with calcium dysregulation and ROS formation in primary neural cells	[24]
18	5–12.50 μg/mL	24 h	3–5	Mouse brain neural cells	AgNPs could alter gene and protein expressions of β-amyloid (Aβ) deposition	[146]
19	800 particles/cell	48 h	20 and 80	Human embryonic neural precursor Cell	AgNPs exposure cause a significant stress response in the growing Human neural progenitor *cells* (*hNPC*)	[147]
20	5 μg/mL	48 h	<100	HT22 mouse hippocampal neuronal cells	AgNPs modulated HT22 cell cycle , proliferation, induced oxidative stress and 53BP1 recruitment	[148]
